# Point prevalence survey of peripheral venous catheter usage in a large tertiary care university hospital in Germany

**DOI:** 10.1186/s13756-019-0468-8

**Published:** 2019-01-17

**Authors:** Seven Johannes Sam Aghdassi, Christin Schröder, Désirée Gruhl, Petra Gastmeier, Florian Salm

**Affiliations:** 1 Charité – Universitätsmedizin Berlin, corporate member of Freie Universität Berlin, Humboldt-Universität zu Berlin, and Berlin Institute of Health, Institute of Hygiene and Environmental Medicine, Berlin, Germany; 2 National Reference Center for the Surveillance of Nosocomial Infections, Berlin, Germany; 3 Institute for Infection Prevention and Hospital Epidemiology, Medical Center, University of Freiburg, Faculty of Medicine, Freiburg, Germany

**Keywords:** Infection control, Bloodstream infection, Sepsis, Phlebitis, Peripheral venous catheter, Healthcare-associated infection, Point prevalence survey

## Abstract

**Background:**

Bloodstream infections (BSI) are among the most frequently documented healthcare-associated infections (HAI). Central and peripheral venous catheters (CVC and PVC) are relevant risk factors for BSI. Although the risk for BSI is higher for CVC, PVC are utilized more frequently and are therefore relevant in the context of HAI prevention. Robust data on the prevalence of PVC and associated infections in German hospitals are scarce to this date. The objectives of this survey were to estimate the prevalence of PVC and PVC-associated infections on peripheral wards of a large tertiary care hospital in Germany. The collected data may be utilized for a tailored infection prevention intervention in the future.

**Methods:**

A point prevalence survey was conducted on peripheral wards of a tertiary care hospital with more than 3.000 beds. Data were collected between August 2017 and February 2018. Standardized data collection forms were used for collecting ward, patient and PVC-related data. As endpoints, prevalence of patients with PVC, PVC-associated infections and PVC without usage in the 24 h prior to the survey and without documentation of intended usage in the 24 h after the survey (“unused PVC”) were chosen. For data analysis, Kruskal-Wallis test was employed for continuous variables and Chi-squared test or Fisher’s exact test for categorical variables. Multivariable analysis and logistic regression were performed for the endpoint unused PVC.

**Results:**

Data from 2.092 patients on 110 wards were collected. The overall prevalence of patients with PVC was 33%. Infections were recorded in 16 patients. Except one case of BSI, these were all local infections at the site of insertion. Of 725 documented PVC, 77 (11%) were unused PVC. Multivariate analysis and logistic regression revealed wards with the practice of regularly obtaining blood from PVC, PVC with dirty or loose insertion dressing, pediatric ward specialty and last inspection of the PVC more than 1 day ago as significant risk factors for unused PVC.

**Conclusions:**

A substantial proportion of patients presented with a PVC on the day of survey. Too few infections were recorded to allow for more detailed analyses. Various risk factors for unused PVC were identified. We hereby present a solid method to obtain an overview about PVC use and to increase awareness for PVC-associated risks. The limitations of point prevalence surveys have to be recognized.

**Electronic supplementary material:**

The online version of this article (10.1186/s13756-019-0468-8) contains supplementary material, which is available to authorized users.

## Background

Healthcare-associated infections (HAI) are a challenge in the practice of modern medicine. The prevalence of HAI in patients hospitalized in German acute care hospitals has been estimated to be approximately 4.6% [1]. A relevant portion of these infections are device-associated [2, 3]. For bloodstream infections (BSI) the most important risk factor is the use of central venous catheters (CVC) and peripheral venous catheters (PVC). The risk for BSI per PVC is lower than per CVC, whereas the application rate of PVC is substantially higher [4, 5], as PVC represent one of the most frequently used medical devices in hospitals [6–8]. Estimations of prevalence rates for PVC range from 24 to 100% across different countries [8]. In Germany, there is a lack of robust published prevalence data concerning PVC use in hospitals. Furthermore, only recently has the collection of data on PVC utilization been introduced as a part of the German national surveillance network (Krankenhaus-Infektions-Surveillance-System, KISS).

The aims of this survey were to estimate the prevalence of PVC and PVC-associated infectious complications, and to assess indicators of PVC management on all wards in a tertiary care university hospital, as a preparation for a tailored infection control intervention to optimize PVC use and management.

## Methods

The point prevalence survey was conducted on peripheral wards of all three campuses of the Charité-University Medicine Hospital in Berlin, Germany, a tertiary care university hospital with more than 3.000 beds and about 150.000 inpatient cases per year.

### Questionnaire

Standardized data collection forms were designed to collect ward, patient and PVC data. On the ward level, the handling of PVC was assessed through a standardized questionnaire to be answered by the head of nursing of the respective ward. Among the data collected were questions exploring which professional groups were generally responsible for PVC insertion, whether PVC were used to draw blood, and whether a catheter extension set was regularly used. The use of such catheter extension sets has been promoted by the local hospital infection control team in recent years. On the patient level, age, sex, the *Physical Status Classification* of the American Society of Anesthesiologists (ASA) score [9], the number of inserted PVC at the time of survey and PVC-associated infections were recorded. Phlebitis was evaluated according to the *Visual Infusion Phlebitis Score* [10], other PVC-associated infections were defined according to the definitions used in the German KISS [11], which are based on the definitions of the United States National Healthcare Safety Network. Patient-related data were collected for all patients, which presented with a PVC on the day of survey as well as for patients with an active PVC-associated infection, even if the PVC had already been removed on the day of survey. An active infection was defined either as fulfilling case definitions on the day of survey or as having fulfilled case definitions prior to the survey and still receiving treatment for the infection, even if symptoms of the infection were no longer present. On the PVC level, the date of insertion, the insertion site, the size of the PVC by Birmingham gauge (G), the kind and condition of the insertion dressing, the last usage of the PVC before the survey and the intended next usage after the survey, whether a catheter extension set was used, which is recommended by the German Commission for Hospital Hygiene and Infection Prevention [12], and indicators concerning PVC documentation were collected. For more details, please find the translated questionnaires in the Additional file 1.

### Data collection

The prevalence survey was conducted on 110 peripheral wards of the Charité-University Medicine Hospital. Intensive care units, intermediate care units and outpatient settings were excluded from the survey. From August 2018 to February 2018 the data collection was executed by a single infection control nurse supervised by two infection control physicians.

### Statistical analysis

Univariable and multivariable analysis were used to compare two groups. Data was subjected to univariable analysis using Kruskal–Wallis-test for continuous data and Chi-squared test for categorical data. When necessary, Fisher’s exact test was used instead of Chi-squared test. In addition, data were analyzed by logistic regression for the outcome whether PVC were still in use (i.e. usage within the 24 h prior to the survey and/or documented intended use in the 24 h after the survey). The following covariates were included: at the ward level: ward specialty, number of beds, percentage of occupied beds, prevalence of patients with PVC on the ward, regular blood sampling via catheter, regular use of a catheter extension set; at the patient level: age, sex, time span between admission to the hospital and survey, time span between admission to the ward and survey, ASA score; on the PVC level: size of PVC, use of a catheter extension set, anatomic location of the PVC insertion site, condition of dressing, and time span between last inspection and survey. Covariates were selected by forward backward selection and included in the model if *p* = 0.05 and excluded from the model if *p* > 0.10. A *p*-value of less than 0.05 was considered significant. Only 696 datasets were included in the logistic regression, 29 datasets were excluded because of missing values.

All analyses were performed using R 3.4.3 (R Foundation for Statistical Computing, Vienna, Austria) and SAS 9.4 (SAS Institute Inc., Cary, NC, USA).

### Ethical approval

All data were anonymized and collected in accordance to paragraph 23 of the German federal law, *German Protection against Infection Act* (“Infektionsschutzgesetz”), which regulates the prevention and control of infectious diseases in humans. Therefore, ethical approval and informed consent were not required.

## Results

**Fig. 1:**
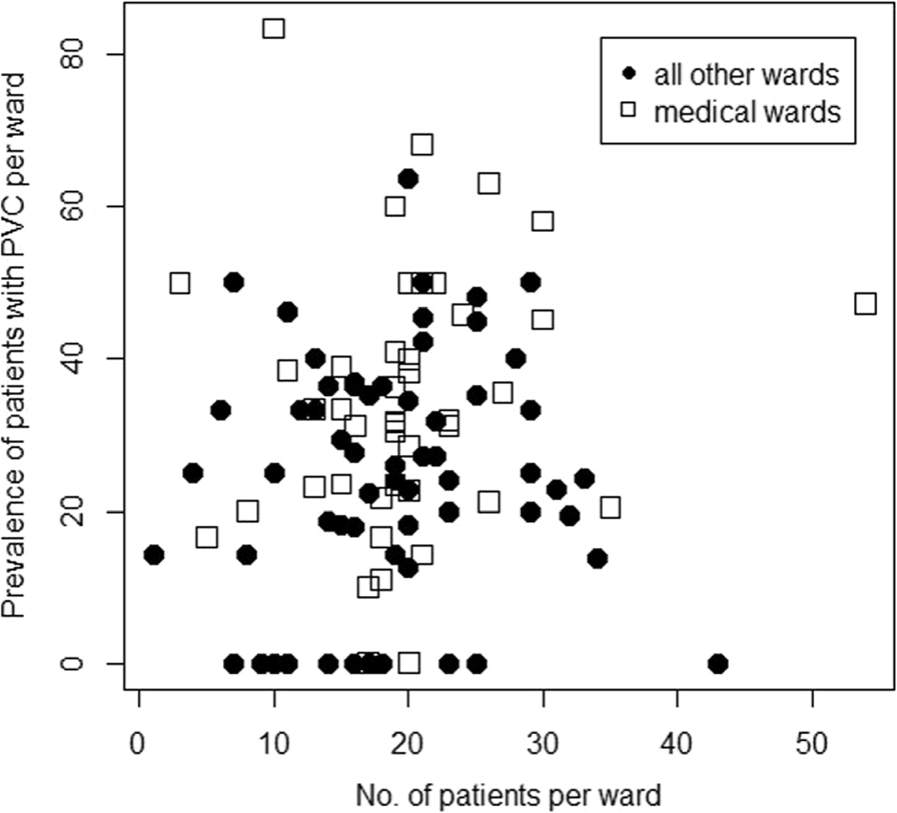
Prevalence of patients with peripheral venous catheters per ward. Scatter plot of prevalence of patients with peripheral venous catheters (PVC) per ward

Data from 2.092 patients in 110 wards were collected between August 2017 and February 2018. 681 of these patients had one or more PVC on the day of survey. The overall PVC prevalence was found to be 33% with substantial differences between wards. Figure 1 illustrates the differences in PVC prevalence between wards. Insertion of PVC was reported by all wards to be done primarily by medical doctors or students of medicine. In 58 wards (53%) PVC were regularly or occasionally used to obtain blood samples from patients. A catheter extension set was reported to be regularly used in 51 (46%) wards.

Patient-related data were collected for a total of 687 patients. The data are summarized in Table 1. Infections were recorded in 16 patients. Ten (63%) of these patients had a PVC on the day of survey. In four patients, the infection was associated with a PVC that was still present on the day of survey. In all other cases, the PVC related to the infection had already been removed (*n* = 6) or had been removed and a new PVC was placed at a different site (*n* = 6). The presence of only local signs of infections at the site of insertion were reported in 15 patients. Four of these patients received conservative local treatment (incl. Cooling and immobilization). In one patient, a PVC-related thrombophlebitis as well as a PVC-related BSI (fulfilling the KISS-definitions for primary sepsis) were recorded. *Staphylococcus aureus* was identified as the causing pathogen. The patient received antimicrobial treatment.

**Table 1:** Patient and peripheral venous catheter-related data

Parameter	Number (percentage)
Patients, total	687
Sex male	360 (52)
Age in years	57^a^
ASA score	2.7^a^
Days of hospitalization until survey	4^b^
Patients with ≥ 1 PVC	681
PVC, total	725
Patients with PVC-related infection(s)	16
PVC with catheter extension set	309 (43)
Site of insertion	
back of hand	267 (37)
forearm	245 (34)
upper arm incl. elbow	204 (28)
other	9 (1)
Size of PVC by Birmingham gauge (G)	
G24/G22	136 (19)
G20/G18	558 (77)
G16/G14	31 (4)
Transparent insertion dressing	725 (100)
Dressing loose or dirty	87 (12)
Days from insertion until survey	2^b^
Last usage before survey in hours	7.2^a^
No documented usage in 24 h prior and after survey	77 (11)
Catheter insertion documented in patient charts	629 (87)
PVC inspected by ward staff on the day of survey or day before	441 (61)

*ASA* Physical Status Classification of the American Society of Anesthesiologists, *PVC* peripheral venous catheter(s); ^a^ mean; ^b^ median

Data on a total of 725 PVC in 681 patients were collected (Table 1). Nine (1%) of 725 PVC showed signs of infection such as pain, induration, redness, and swelling. No visual phlebitis score higher than two was recorded in the survey.

Univariable analysis focusing on risk factors for PVC without usage in the 24 h prior and after the survey (referred to as “unused PVC”) revealed pediatric ward specialty, the practice of regularly using PVC for blood sampling, small size of the PVC, bad condition of the insertion site dressing as well as a long interval since last inspection as significant risk factors of unused PVC. The use of a catheter extension set was identified as decreasing the likelihood of unused PVC (Table 2).

**Table 2:** Univariable analysis of risk factors for peripheral venous catheters without usage in the 24 h prior and after survey

	PVC with usage (*n* = 627) number (percentage)	PVC without usage (*n* = 69) number (percentage)	*p*-value
PVC regularly used for blood sampling on ward	373 (59)	59 (86)	< 0.05
Regular use of catheter extension set on ward	331 (53)	19 (28)	< 0.05
Ward specialty			< 0.05
Medical^a^	307 (49)	20 (29)	
Surgery^b^	119 (19)	16 (23)	
Other surgery^c^	134 (21)	17 (25)	
Interdisciplinary/other	38 (6)	8 (12)	
Pediatrics	29 (5)	8 (12)	
Number of beds on ward > 22 (median)	303 (48)	28 (41)	0.27
Percentage of occupied beds per ward (mean)	88.6	88.6	0.94
Prevalence of patients with PVC on ward (mean)	43.5	44.2	0.58
Sex, male	331 (53)	33 (48)	0.51
Age (in years)			0.86
0–18	32 (5)	4 (6)	
19–35	90 (14)	12 (17)	
36–55	119 (19)	10 (14)	
56-70	178 (28)	19 (28)	
> 70	208 (33)	24 (35)	
Hospital stay > 7 days	168 (27)	14 (20)	0.30
Stay on ward > 7 days	121 (19)	10 (14)	0.41
ASA score			0.09
1–2	130 (21)	21 (30)	
3–5	497 (79)	48 (70)	
Insertion site			0.34
Back of hand	225 (36)	30 (43)	
Forearm	211 (34)	25 (36)	
Upper arm incl. elbow	186 (30)	14 (20)	
Other	5 (1)	0 (0)	
Size of PVC by Birmingham gauge (G)			< 0.05
G24/G22	126 (20)	5 (7)	
G20/G18	477 (76)	59 (86)	
G16/G14	24 (4)	5 (7)	
Dressing loose or dirty	65 (10)	20 (29)	< 0.05
Days since last inspection			< 0.05
0	213 (34)	2 (3)	
1	394 (63)	46 (67)	
> 1	20 (3)	21 (30)	
PVC with catheter extension set	292 (47)	19 (28)	< 0.05

*PVC* peripheral venous catheter(s); ^a^incl. internal medicine, dermatology, neurology, geriatrics; ^b^traumatology and abdominal surgery; ^c^incl. urology, gynecology, otolaryngology

Logistic regression as illustrated in Table 3 revealed that patients in pediatric wards had a significantly higher risk of having an unused PVC than patients in medical wards. Differences were also found when comparing medical wards to wards of other specialties, however, these were not significant. Furthermore, logistic regression identified patients on wards, where PVC were regularly used for obtaining blood samples to have a significantly higher risk of having an unused PVC, than patients on wards where PVC are rarely or never used for drawing blood. A dirty or loose insertion dressing was revealed to be a significant risk factor for unused PVC by multivariable analysis. Inspection on the day of survey or within the 24 h prior significantly reduced the risk for unused PVC when compared with inspection more than 2 days before the survey.

**Table 3:** Logistic regression model of risk factors for peripheral venous catheters without usage in the 24 h prior and after survey

	Odds Ratio (95%-confidence interval)	*p*-value
PVC regularly used for blood sampling on ward		< 0.05
No	reference	
Yes	3.37 (1.60–7.10)	
Days since last inspection		< 0.05
2–8	reference	
0	0.01 (0.00–0.04)	
1	0.13 (0.06–0.28)	
Ward specialty		< 0.05
Medical^a^	reference	
Surgery^b^	1.26 (0.57–2.74)	
Other surgery^c^	1.32 (0.62–2.84)	
Interdisciplinary/other	2.08 (0.77–5.64)	
Pediatrics	10.41 (2.71–40.07)	
Condition of insertion dressing		< 0.05
Dirty or loose	reference	
Well-maintained	0.25 (0.13–.0.48)	

*PVC* peripheral venous catheter(s); ^a^incl. internal medicine, dermatology, neurology, geriatrics; ^b^traumatology and abdominal surgery;^c^ incl. urology, gynecology, otolaryngology

## Discussion

We analyzed data of over 2.000 patients. Approximately one in three patients (681) presented with one or more PVC on the day of the point prevalence survey. A similar prevalence was reported in a study in the United Kingdom by Reilly et al. [13]. In our survey too few infections were recorded to allow for detailed analyses of risk factors. In total, PVC-related infectious complications were found in very few of the recorded PVC. Except one case of PVC-related BSI, these were all local infections, ranging from redness and swelling to signs of thrombophlebitis. These findings indicate that PVC at our hospital, from the perspective of an individual patient, pose a low risk for catheter-related BSI or other severe infectious complications. Other studies have yielded similar results [14]. The insertion of PVC is one of the most common invasive procedures in hospitals [6, 15, 16]. The BSI infection rate per 1.000 PVC device-days is estimated to be around 0.6 (95CI 0.2–0.9) [17]. Therefore, due to the frequency of application and severity of possible complications the topic gains very high relevance from a public health perspective.

A substantial proportion of PVC were identified as unused PVC on the day of survey. On wards, where the healthcare personnel regularly obtained blood from PVC, a significantly higher number of unused PVC were recorded. These unused PVC were less likely to be inspected on the day of survey or the day before and therefore can be regarded as an unnecessary infection risk for the patient in two respects: first, the mere presence of an invasive device that might no longer be needed; second, the lack of inspection of the device. Our method of data collection did not allow for differentiating whether an inspection, that was carried out, was simply not documented or whether no inspection was undertaken in the first place. Both possibilities, however, illustrate the need for improving systematic PVC management and documentation. The fact that multivariable analysis revealed a loose or dirty (i.e. not well-maintained) insertion dressing to be a risk factor for unused PVC, corroborates this result.

It remains unclear whether the high number of unused PVC (as defined by the above-stated criteria) were simply forgotten by the ward staff or intentionally remained inserted to be available in case the patient’s condition changed, and an intravenous application of fluids or medication became necessary again. The practice of leaving PVC inserted “just in case” is a phenomenon that was previously described by other authors [18–20]. These studies and our data emphasize the importance of a daily, systematic, and documented inspection of PVC which should be an integral part of good clinical practice [12, 21].

Education and feedback of PVC-related surveillance data or topics have frequently been cited as effective intervention strategies to improve PVC use and management [21–23]. However, the basis of education and feedback are information such as the data collected in the present survey. This kind of data collection is intensive in time and personnel expenditure. Other intervention strategies such as checklists or daily reminder systems may be effective as well, and easier to establish [24–26]. Interestingly, multivariate analysis revealed that the practice of regularly using catheter extension sets significantly decreased the probability of unused PVC. This can be seen as an indicator that wards which have a high educational level with regard to PVC management are more likely to realize timely catheter removal, thus, demonstrating the efficacy of the above-mentioned intervention strategies.

Patients in pediatric wards were identified by multivariable analysis to have a significantly higher risk for unused PVC than patients in medical wards. This is especially critical since these patients represent a particularly vulnerable population. Univariate analysis showed that smaller PVC (G24/G22) were a risk factor for unused PVC as well. Since smaller PVC are inserted more frequently in pediatric wards, this finding can be interpreted as a consequence of the effect of the ward specialty.

Point prevalence surveys provide a solid method to obtain an overview and to identify areas of potential improvement. Importantly, it has to be recognized that this method entails relevant limitations. Among these are:

- Only PVC present at the time of survey were recorded. PVC which may have been present in the days before conducting the survey and which had been removed before data collection were not recorded. Therefore, we cannot make an accurate statement on the frequency of PVC usage.
- Only PVC-associated infections which were active at the time of survey were recorded. Infections occurring before or after the survey could not be recorded with the methodology applied. Furthermore, patients with severe infectious complications, such as BSI, may have been transferred to intensive care units. Since data collection was conducted on peripheral wards only, such infections would have been missed. Therefore, accurate estimations on the burden of PVC-associated infections are not possible with the data collected in the survey present.
- Ward data was collected through a systematic questionnaire to be answered by the head nurse. These answers may be subjective in some cases.
- Reluctance by ward staff to collect microbiological specimen in case of suspected infection may lead to an underestimation of PVC-related infections.
- The present survey is a single-center survey and cannot be used for extrapolations on a national level.
- Patients with longer hospital stays are generally overrepresented in point prevalence surveys.

A strength of the survey is that it represents a true cross-section of the included peripheral wards of our hospital since all patients of these wards were assessed. All data were collected by a single infection control nurse, experienced and well-trained in aspects of surveillance and data collection. We thereby were able to reduce inter-rater bias to a minimum.

## Conclusion

Our survey demonstrated that the duration of insertion of PVC is not only determined by how long they are utilized for intravenous application, but also by other factors. Some of these factors appear to be structural or organizational, such as the practice of regularly using PVC for obtaining blood samples. It appears that PVC may remain inserted longer because of convenience reasons, thereby putting patients at an unnecessary risk for PVC-related complications. Lower frequency of PVC inspection seems to contribute to this negative effect. Feasible intervention strategies generated from the data presented here, may be to feed back the data to clinicians and relevant stakeholders, to organize targeted education and training programs and to develop checklists and reminder systems to decrease the number of unnecessary PVC and device-days.

## Additional file

Additional file 1: PVC_survey_questionaires_english_version. (DOCX 14 kb)

## Abbreviations

ASA: American Society of Anesthesiologists
BSI: Bloodstream infection(s)
CVC: Central venous catheter(s)
G: Birmingham gauge
HAI: Healthcare-associated infection(s)
KISS: Krankenhaus-Infektions-Surveillance-System
PVC: Peripheral venous catheter(s)
