# Supplementary material for: Point prevalence survey of peripheral venous catheter usage in a large tertiary care university hospital in Germany
Source: Antimicrob Resist Infect Control. 2019 Jan 17;8:15. doi: 10.1186/s13756-019-0468-8 (PMC6335674; doi:10.1186/s13756-019-0468-8)
Supplement: Supplementary file 1 — PVC_survey_questionaires_english_version. (DOCX 14 kb) [file 13756_2019_468_MOESM1_ESM.docx]

**Data collection sheet 1 – WARD DATA –**

To be filled out once for every ward included

1. Date: _____/______/_____

2. Ward-ID (anonymized):_________

3. Ward-specialty:______________

4.-7. Number of:

| Patient beds | Patients present | Patients present with ≥ 1 PVC | Patients with active PVC-associated infection |
| --- | --- | --- | --- |
|  |  |  |  |

*PVC* Peripheral venous catheter(s)

8. Insertion of PVC primarily done by

[ ] Medical doctors [ ] Nursing staff [ ] Students of Medicine [ ] Other staff

9. How frequently are PVC used for obtaining blood samples?

[ ] Regularly [ ] Rarely [ ] Never

10. Is a catheter extension set regularly used?

[ ] Yes [ ] No

**Data collection sheet 2 – PATIENT DATA –**

To be filled out once for every patient with a PVC and/or active PVC-related infection

1. Patient-ID (anonymized): _________

2. Age:____ 3. Sex: [male] [female] [other]

4. Date of hospital admission:___/___/ ___ 5.Date of ward admission___/__/___

6. ASA score : [ASA-1] [ASA-2] [ASA-3] [ASA-4] [ASA-5] [ASA-6]

*ASA* Physical Status Classification of the American Society of Anesthesiologists

7. Number of PVC, which the patient presents at the time of survey ____

*PVC* Peripheral venous catheter(s)

8. Has the patient a PVC-associated infectious complication?

[ ] Yes (->continue with questionaire) [ ] No (-> end of questionaire)

9. Which PVC-associated infectious complication does the patient have? (more than one answer possible)

[ ] Local infection (not fulfilling KISS-definitions)

[ ] Fever (>38° C)

[ ] Hypotension (< systolic 90 mm/HG)

[ ] Thrombophlebitis

[ ] L2 (see KISS-definitions)

[ ] F1 (see KISS-definitions)

[ ] B1 (see KISS-definitions)

*KISS* Krankenhaus-Infektions-Surveillance-System

10. Is/are the complication(s) listed in 9 associated with a currently inserted PVC?

[ ] Yes [ ] No

11. If applicable, which microorganism was identified as the causing pathogen and from which material?

_________________________________________________________

12. Does the patient receive treatment for the complication(s) listed in 9? [ ] Yes [ ] No

13. If yes, what kind of treatment is the patient receiving? (more than one answer possible)

[ ] Systemic antibiotic therapy

[ ] Local antibiotic therapy

[ ] Local therapy (non-antibiotic)

[ ] Surgical intervention

[ ] Conservative therapy (cooling, immobilization)

[ ] Other (please specify) __________________

**Data collection sheet 3 – PERIPHERAL VENOUS CATHETER DATA –**

To be filled out once for every peripheral venous catheter (PVC)

1. PVC-ID:________________

2. Date of insertion: ____/____/____

3. PVC size by Birmingham gauge (G): _______

4. Does the PVC have a catheter extension set? [ ] Yes [ ] No

5. Insertion site (anatomical location): _______

6. Kind of insertion dressing: [ ] Transparent [ ] Non-transparent [ ] Other (specify)

7. Insertion dressing loose? [ ] Yes [ ] No

8. Insertion dressing dirty? [ ] Yes [ ] No

PVC documentation:

9. Documentation of catheter insertion in patient files? [ ] Yes [ ] No

10. Time and date of last documented inspection _______________________

PVC usage:

11. Time and date of last documented usage _______________________

12. Time and date of next intended usage _______________________

PVC complications:

13. Clinical signs of infection? [ ] Yes [ ] No

14. Pain at insertion site? [ ] Yes [ ] No

15. Pain on palpation? [ ] Yes [ ] No

16. Swelling? [ ] Yes [ ] No

17. Induration of soft tissue? [ ] Yes [ ] No

18. Reddening at insertion site? [ ] Yes [ ] No

19. Purulent drainage? [ ] Yes [ ] No
